# A Method for Evaluating the Efficacy of Antifouling Paints Using *Mytilus galloprovincialis* in the Laboratory in a Flow-Through System

**DOI:** 10.1371/journal.pone.0168172

**Published:** 2016-12-13

**Authors:** Ryuji Kojima, Seiji Kobayashi, Cyril Glenn Perez Satuito, Ichiro Katsuyama, Hirotomo Ando, Yasuyuki Seki, Tetsuya Senda

**Affiliations:** 1 Department of Marine Environment and Engine System, National Maritime Research Institute, Mitaka, Tokyo, Japan; 2 Department of Environmental Risk Consulting, Japan NUS Co., Ltd, Shinjuku, Tokyo, Japan; 3 Graduate School of Fisheries Science and Environmental Studies, Nagasaki University, Nagasaki, Japan; 4 Hiroshima R&D Centre, Chugoku Marine Paints, Ltd, Otake, Hiroshima, Japan; VIT University, INDIA

## Abstract

A laboratory test with a flow-through system was designed and its applicability for testing antifouling paints of varying efficacies was investigated. Six different formulations of antifouling paints were prepared to have increasing contents (0 to 40 wt.%) of Cu_2_O, which is the most commonly used antifouling substance, and each formulation of paint was coated on just one surface of every test plate. The test plates were aged for 45 days by rotating them at a speed of 10 knots inside a cylinder drum. A behavioral test was then conducted using five mussels (*Mytilus galloprovincialis*) that were pasted onto the coated surface of each aged test plate. The number of the byssus threads produced by each mussel generally decreased with increasing Cu_2_O content of the paint. The newly designed method was considered valid owing to the high consistency of its results with observations from the field experiment.

## Introduction

Antifouling (AF) paints should be evaluated and selected from the viewpoint of fuel consumption efficiency and effectiveness in preventing the attachment of marine fouling organisms on ships [1–5]. However, the attachment of unwanted aquatic species is strongly influenced by region, season [6–8], and usage of vessels [9–11], and suitable applications of AF paints rely on expert recommendation. Presently, the evaluation of new AF paints is conducted by paint manufacturers according to their respective protocols. The results of their evaluation are classified hence there are no objective data on the efficiency of antifouling paints. The controlled laboratory conditions during tests of AF paints do not accurately reflect those in the natural environment where biofouling occurs on ship hulls [12].

AF paints have been evaluated in the past mostly through field experiments on rafts and by patch-tests on ship hulls. For instance, the Efficacy Assessment Guideline of the European Chemical Agency (ECHA) clearly states that field experiments are necessary to evaluate efficiency under natural conditions where biofouling occurs [13]. In this manner, the common practice in evaluating the efficiency of AF paints is the wait-and-see approach, which involves immersing AF paint coated test plates by hanging them from a raft and observing biofouling [14–17]. In raft trials conducted in coastal waters, conditions of the marine environment (i.e., seasonal variation, geographical factor, etc.) influence test results [18]. Consequently, results of the control group may not exhibit a constant trend in response to evaluation criteria, such as biomass occurrence of biofouling species. Hence, it becomes difficult to make an objective evaluation of the efficiency of AF paints by comparing control and experimental groups. Also, field experiments on rafts usually need varying numbers of test plates and a longer time to obtain results, making them costlier to perform [19]. Accordingly, biofouling data obtained from raft trials conducted at different sites and in different seasons exhibit large variations [20–26], making it insufficient to conduct an objective and quantitatively evaluation of various AF paints. In this regard, it is necessary to establish a bioassay to assess AF paints under controlled laboratory conditions. On the other hand, the efficiencies of new antifoulants derived from either natural products or by artificial synthesis have been assessed through inhibition and toxicity assays where the experimental system has a still water condition [12, 27]. Therefore, in efficiency tests of AF paints, it is important to establish a controlled experimental condition to ensure reproducible results.

A wide range of test organisms has been used in AF bioassay in controlled experimental conditions utilizing dominant macrofoulers. Mussels are one of the dominant macrofoulers and are recorded as major fouling organisms of marine structures and ships’ fouling, hence they are used in settlement inhibition assays [12]. In mussel experiments, the settlement stage of larvae called pediveligers [27–30] and settled young or adult mussels are used as test organisms. In this regard, the authors used young mussels for the purpose of this experiment. The antifouling effect on mussels by the byssus threads production of adults fixed on substrates was evaluated [31], while others made evaluations from the selective behavior of freely moving young individuals [32–36]. The method by Harada [31] was adopted in this study because it does not require numerous numbers of test panels to conduct the evaluation. This method was also adopted to study mussel attractants [37] and antifouling effect of metals [38].

In this paper, the authors prepared test plates coated with different AF paints and tested their antifouling efficiencies in a newly proposed laboratory-based method under controlled conditions using mussels, *Mytilus galloprovincialis*. The purpose was to confirm whether the efficiency of the AF paints was directly reflected in an appropriate manner in the newly proposed evaluation method. The AF paints tested in the experiment contained only cuprous oxide (Cu_2_O) as the antifoulant, and had increasing Cu_2_O contents. The test plates coated with the test AF paints were aged dynamically in a manner that their surface simulated the real condition of the ships’ hull. After aging, the test plates were then used in the mussel settlement assay. The efficiencies of AF paints were evaluated by the number of byssus threads secreted by the mussels fixed on the test plates. The proposed evaluation method was validated by comparing results of laboratory experiments with antifouling efficiency results from field experiments on rafts.

## Materials and Methods

### AF paints and test plates

#### Compositions of AF paints

The matrix polymer of the paints tested was a self-polishing hydration type copolymer that was polymerized by vinyl chloride and isobutyl vinyl ether. Cu_2_O was the only antifouling agent used in the test paints, because it enabled easier analysis and evaluation of the leaching phenomenon, and also eliminated the synergy effect between Cu_2_O and other booster biocides [39]. Six AF paints with 0, 5, 10, 20, 30 and 40 wt. % of Cu_2_O were prepared for the experiment. The pigment concentration in the paints was kept constant by adjusting the contents of the extender pigment and resin. Compositions of the test paints are shown in Table 1.

**Table 1:** Compositions of the test paints used in the study.

Composition ^a)^ / Paint name	A-0	A-1	A-2	A-3	A-4	A-5
Cuprous Oxide	0	5	10	20	30	40
Xylene	23	23.6	24	25	26	27
Methylisobutylketone	5	5	5	5	5	5
Base polymer	9	8.7	8.5	8	7.5	7
Rosin	9	8.7	8.5	8	7.5	7
Barium sulfate	50	45	40	30	20	10
Anhydrous ferric oxide	1	1	1	1	1	1
Oxidized polyethylene wax	1	1	1	1	1	1
Amide wax	2	2	2	2	2	2

a) Values in the Table indicate mass %.

Polyvinyl chloride (PVC) plates (50 mm x 50 mm x 2mm) that were not coated with the test paint were used as the control plates in laboratory experiments. PVC control plates (150 mm x 100 mm x 5mm) that were used in the field experiment were also not coated with test paints, but their surfaces were scrubbed with sandpaper (#120) prior to use. For the experimental plates, PVC plates were used in the laboratory experiments, while sandblasted steel plates (Sa 2.5) were used in field experiments. These test plates had the same size as their control counterparts, and were coated with test paints on only one side. These test plates were coated first with 100 μm thickness of epoxy resin, then with a second coating of 100 μm thickness of epoxy binder, and dried at room temperature for 20 h. The dried test plates were further twice coated with 50 μm thickness of the test paints, and finally dried for 7 days at room temperature [40].

#### Dynamic aging of the test plates in laboratory

**Fig 1:**
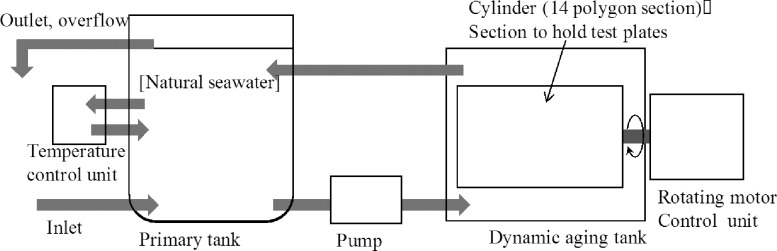
A schematic diagram of the dynamic aging system.

**Fig 2:**
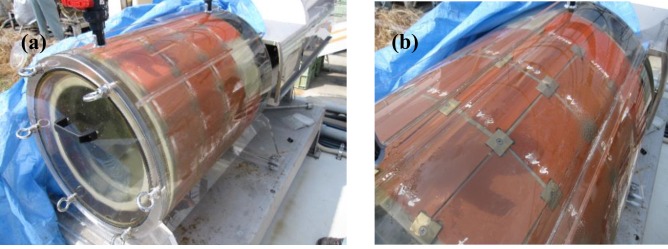
Photographs of the dynamic aging apparatus. (a) the cylinder with test plates fixed on it, and (b) the test plates inside the cylinder.

The novel dynamic aging system consisted of a water tank (ca. 230 L) installed with an apparatus to hold the test plates, a thermostat that kept the temperature constant at 20±3°C, a controller and a primary storage tank (ca. 300 L, the replenishing rate: ca. 0.7 L/min.), as shown in Fig 1. The water tank for dynamic aging was a concentric cylindrical structure. The apparatus that held the test plates for laboratory experiment was a 14 polygon section cylinder, as shown in Fig 2.

During the aging process, the cylinder apparatus holding the test plates was rotated by a motor at a plate surface speed of 5 m/sec (ca. 10 knots) for 45 days with water flow rate of 50 L/hr. This aging process was equivalent to 20,000 km of travel distance by oceangoing vessels. During its operation, the water inside the apparatus was always renewed by the continuously flowing filtered seawater in it, thereby preventing leached Cu_2_O from accumulating inside the apparatus and being re-absorbed into the surface of the test plates. After the aging process, the surfaces of test plates were covered with microbial biofilm. Microbial biofilm can affect the leaching rate of antifouling agents [41, 42], but has an inductive effect on the settlement of mussels [43–48]. Hence, the film was gently wiped off the surface of the test plates prior to use in laboratory experiments.

#### Measurement of Cu_2_O concentration in the test water of the laboratory experiment

The concentration of Cu_2_O in the test water of each experimental group was measured after each experiment. Ten ml of the test solution was first desalted by solid-phase extraction method (Inert SEP ME-2, GL Science), and its Cu_2_O concentration was determined using ICP-MS (Agilent 7500i) after adjusting the amount of the solution to 10 ml by adding ultrapure water (Milli-Q system, Merck Millipore).

### Laboratory experiment of bioassay

#### Test apparatus and conditions

Seven-liter glass tanks were used in laboratory experiments. These tanks were each filled with 2 L of the test seawater prior to the experiments. During experiments, 1 μm mesh filtered test seawater was continuously pumped (14ml / min.) into the tank from the test seawater storage tank in a laboratory using a peristaltic pump. A drainage system was attached to each tank to allow water in the tank to flow out through a siphon tube. The rate of test seawater exchange inside the tank was adjusted to ca. 10 exchanges / day. Nine replicate tanks were prepared for one experimental group. Experiments were conducted in an incubator with a 12 h light: 12 h dark environment at a 2000 lux light intensity during the light period, simulating a light/ dark period where actual ships’ hulls are exposed. Water temperature, pH and salinity of the test water in the tanks were measured at the start and 24 h after experiment.

#### Culture of mussels

No specific permits were required to collect mussels in the study sites. *Mytilus galloprovincials* is excluded in the list of regulated living organisms under the invasive alien species act by Ministry of the Environment Government of Japan [49]. Mussels *M*. *galloprovincialis* with shell lengths ranging from 7.6 to 11.2 mm were collected from ropes attached to floating stages off the coast of Yokosuka in Tokyo bay (35°16'N, 139°41'E). These mussels were acclimated 7 days before the start of the experiment at 20°C±2°C in a 12 h light (3000 lux): 12 h dark environment and were fed daily with *Chaetoceros gracilis*. When the difference between the experimental water temperature and that of the sampling site exceeded 5°C, mussels were initially cultured at the same water temperature as that of the sampling site; and the temperature was gradually increased by 1°C / day and acclimatized at 20°C. The seawater used was the same as that used in the experiments, which was 1 μm mesh filtered seawater with a salinity adjusted to 30‰ by diluting it with purified water. Only mussel groups having survival rates higher than 95% during the acclimation period were used in the experiments.

#### Enumeration of byssus threads production of mussels

**Fig 3:**
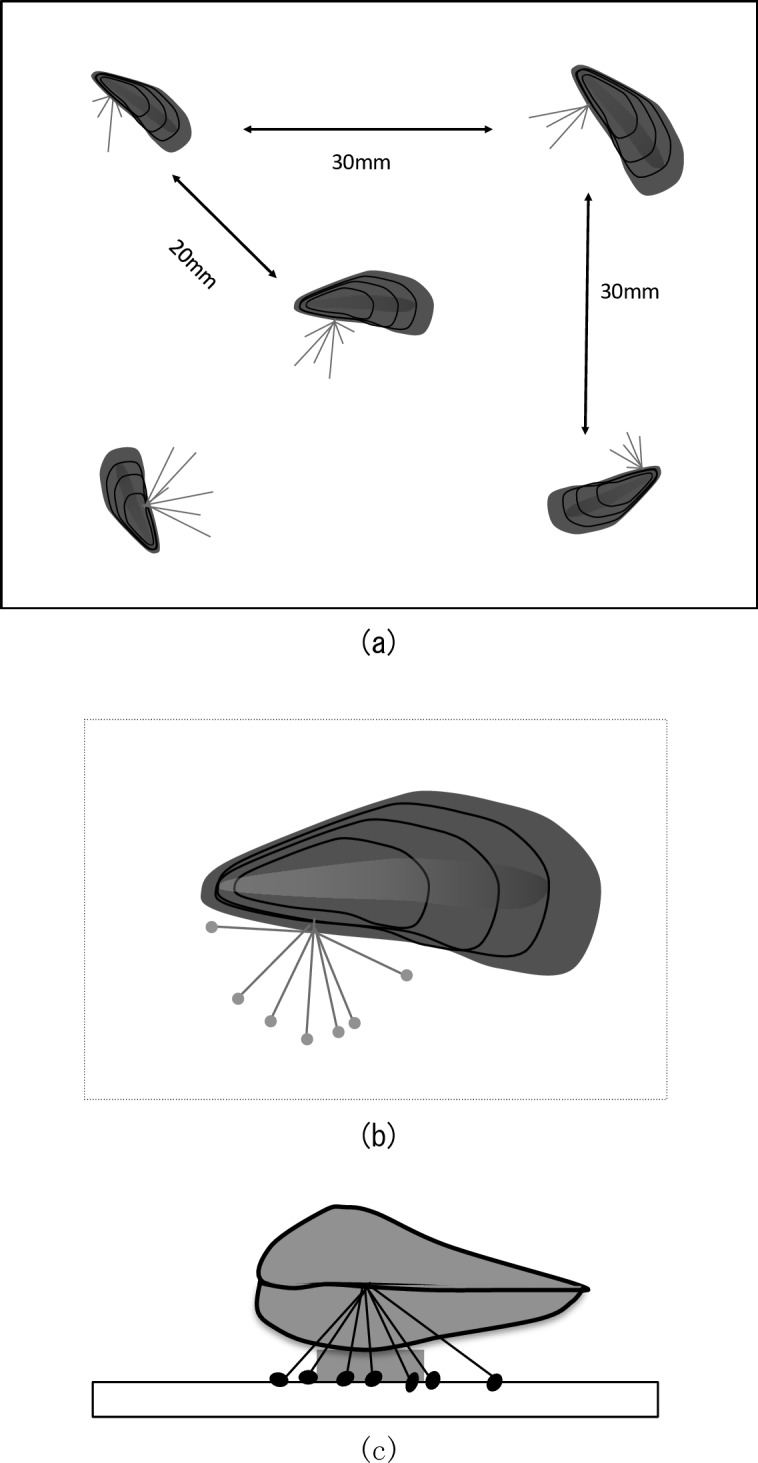
A diagram of a test plate with mussels fixed on its surface. (a), the position of five mussels fixed on the test plate; (b), top view of an individual (enlarged) with its byssus threads and attachment plaques; and (c), side view of an individual fixed on top of a filter paper glued to the test plate.

During acclimation, the shell movements of living mussels were observed, and were selected for the experiments. Mussels were detached from the substrate by carefully cutting their byssus threads with scissors, making sure that tissues connected to the byssus threads were not damaged. Five mussels were fixed to the surface of each test plate (50 mm x 50 mm x 2 mm), with their body axes parallel to the surface. A cyano-acrylate adhesive (Aron Alpha A, surgical grade, Daiichi Sankyo) was used to fix the mussels to the test surface. A diagram of a test plate with mussels fixed on its surface is shown in Fig 3. Each test plate was placed at the bottom of the glass tank and incubated for 24 h. After incubation, test plates were removed from the water tanks and byssus threads produced by individual mussels were counted under a microscope. Attachment plaques were also counted as byssus threads when these were attached to the test plate, even if these were cut off from the individual. Therefore, the number of attachment plaques was taken into account in the calculation of the byssus thread production. The mortality of mussels was also checked. Nine replicate experiments were conducted in October, November and December of 2013.

#### Evaluating the inhibiting effect of test plates using byssus threads production of mussels as a standard

A total of nine experiments on byssus threads production of mussels were conducted; three times each in October, November and December 2013. In each experiment, three test plates for the control and one test plate each for the six experimental groups were evaluated, with each test plate having 5 mussels fixed to it. Accordingly, a total of 405 mussels were used in the nine experiments; 135 individuals in the control groups and 270 individuals in the experimental groups. No mortality was observed throughout the experiments and less than one individual per group was observed detached in the control and experimental groups.

The inhibiting effect of Cu_2_O on the surface of the test plate was evaluated by comparing the number of byssus threads between the experimental and control groups. The average number of byssus threads produced by an individual mussel in the control group was calculated according to Eq 1:

$$A_{c}=\frac{1}{n}\sum_{i=1}^{n}N_{ci}$$

where *N*_*ci*_ indicates the number of byssus threads produced from alive and adhered individual mussel on the control plate; *n*, the number of alive and adhered individual mussel on the control plate; and *A*_*c*_, the average number of byssus threads in the control round.

The average number of byssus threads produced by an individual mussel in experimental group is calculated according to Eq 2:

$$A_{e}=\frac{1}{n}\sum_{i=1}^{n}N_{ei}$$

where *N*_*ei*_ indicates the number of byssus threads and attachment plaques produced from alive and adhered individual mussel on the test plate; *n*, the number of alive and adhered individual mussel on the test plate; and *A*_*e*_, the average number of byssus threads in the experimental round (n).

The ratio of byssus threads production (R), which is the average number of byssus threads produced in the experimental group in comparison to that of the control group, was calculated according to Eq 3:

$$R=\frac{A_{e}}{A_{c}}\times 100$$

#### Field experiment

**Fig 4:**
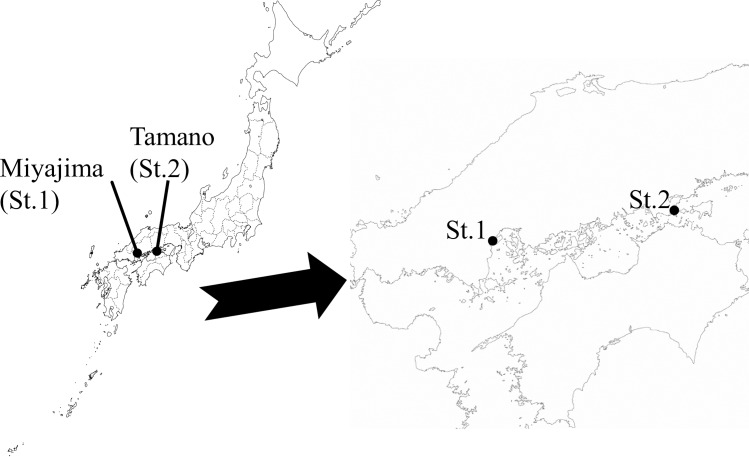
Map showing the sites of the raft trial experiments The source of the map is provided for free from http://www.freemap.jp/item/japan/japan2.html, http://www.freemap.jp/item/region/tyugoku_shikoku.html.

Field experiments were conducted in August (Round-1, summer), October (Round-2, autum), December (Round-3, winter) of 2013, and in May 2014 (Round-4, spring) at Miyajima (Hiroshima Prefecture, Japan: St.1, 34°15'N, 132°15'E) and Tamano (Okayama Prefecture, Japan: St.2, 34°31'N, 133°59'E) experimental sites, located in the inland sea of Japan, as shown in Fig 4.

Test plates were coated using the same test paints shown in Table 1, and fixed onto test racks. The test racks were hung from rafts at the experimental sites and immersed in the sea at a depth of 50 cm from the sea surface to the head of the rack, and the three test racks were 1 m apart from each other. The test plates were immersed in a position perpendicular to the sea surface, and two replicate test plates were immersed for each experimental group. The immersion period at both experimental sites was 28 days, after which biofouling on the test plates was evaluated. Repetitions of trials were sufficient and were more than in the method proposed by CEPE (European Council of the Paint, Printing and Artists’ Council) [17]. Biofouling was evaluated by ranking the degree of fouling as: 0, clean (without biofouling); 1, low fouling (< 0.3%); 2, slightly fouled (< 1%); 3, moderately fouled (< 3%); 4, fouled (<10%); 5, heavily fouled (> 10%); through visual and tactile examination. The degree of fouling was determined according to the percentage of the area of the test plate that was occupied by fouling organisms. No specific permits were required to conduct the field studies in the study sites. The field studies did not involve endangered or protected species.

### Statistical analysis

Statistical analysis including one-way analyses of variance (ANOVA), nonparametric tests, Kruskai-Wallis test (p<0.05), and calculation of correlation coefficients, were conducted using PRISM version 6.0h (GraphPad Software, 2015).

## Results

### Evaluation of the efficacy of AF paint using byssus threads production of mussels

#### Water quality parameters of the test water

The water temperature, salinity and pH of the test waters in the nine experiments were controlled at 20.3 ± 0.6°C, 30.6 ± 0.4 ‰ and 8.2 ± 0.1, respectively. The concentrations of Cu_2_O in the test waters of the control groups ranged from 0.2 μg/L to 0.8 μg/L in the three experiments. On the other hand, the concentrations of Cu_2_O in test waters of the experimental groups were 4.5 μg/L, 9.6 μg/L, 17.9 μg/L and 21.2 μg/L in the groups with 5 wt.% Cu_2_O content (A-1), 10 wt.% Cu_2_O content (A-2), 20 wt.% Cu_2_O content (A-3) and 30 wt. % Cu_2_O content (A-4), respectively, in the December 2013 experiment. The concentrations of Cu_2_O in the test waters of the 40 wt. % Cu_2_O content (A-5) groups ranged from 22.4 μg/L to 42.2 μg/L in the three experiments. Results indicate that the amount of Cu_2_O elution in the test water increased with increasing Cu_2_O content of the paints.

The concentration in the dynamic aging tank was 26 ppb (avg., n = 3) after dynamic aging. Furthermore, at the 0 wt. % Cu_2_O content group, the authors confirmed that the concentration after bioassay was almost the same as with the level in natural seawater (< 1 μg/L). The leaching rate of each panel was confirmed to be at a steady state after 45 days. These results suggest that there was no absorption from the water in the dynamic aging tank, and no subsequent leaching from the paint surface. Therefore, the authors concluded that the concentration build-up of Cu_2_O had no effect on the subsequent leaching from the surface of the test panels.

The number of byssus threads produced by the mussels differed in each of the control groups of the nine experiments conducted in October, November and December. This result is consistent with previous reports [50, 51], where mussels exhibited considerable individual variability in byssus threads production. In order to obtain the repellant effect of paints, data obtained were normalized by calculating the ratios of byssus threads production (R) of mussels in the experimental groups to their respective controls.

**Fig 5:**
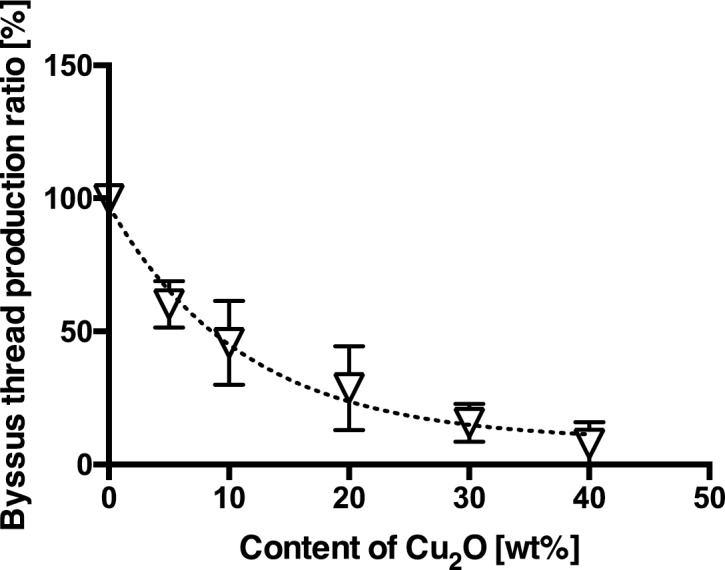
The relationship between byssus threads production ratio and content of Cu_2_O. The byssus threads production ratios in relation to the concentration of Cu_2_O in the paints, obtained in October, November and December 2013. The error bars on the open triangles indicate the SD in the ratio of byssus threads production.

The R values for the paints containing different concentrations of Cu_2_O are shown in Fig 5. R values decreased with increasing Cu_2_O content in the paint used, indicating a clear relationship (r^2^ = 0.9118, non-linear curve fitting, one-phase decay) between R and the Cu_2_O contents in the paints. The results also showed that the R value at 10 wt. % of Cu_2_O content was approximately 50% and byssus threads production in this experimental group obviously differed from the control group. That is, 50% and less R values can be considered as inhibition of byssus threads production.

### Field experiment

**Fig 6:**
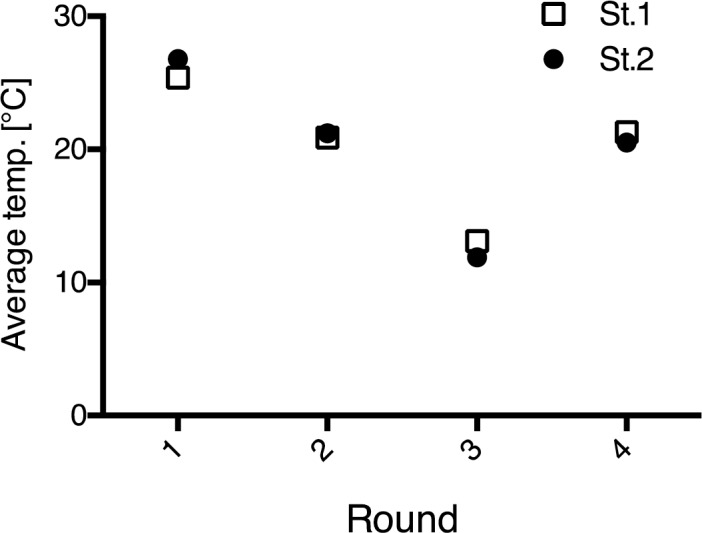
Average seawater temperatures in each of the four rounds conducted at Miyajima (St.1: □) and Tamano (St.2: ●) experimental sites.

**Fig 7:**
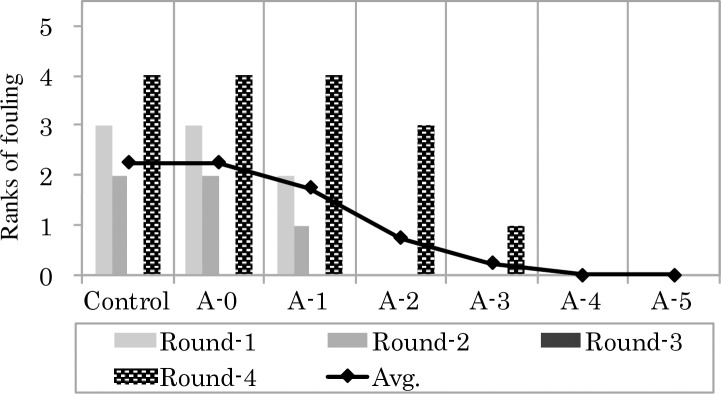
Ranks of fouling by marine animals on the test plates submerged at St. 1.

**Fig 8:**
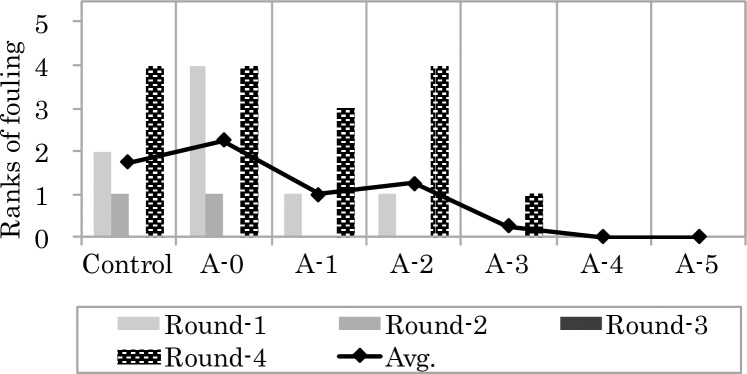
Ranks of fouling by marine animals on the test plates submerged at St. 2.

Average seawater temperatures and results of the field experiments at Miyajima (St.1) and Tamano (St.2) experimental sites are shown in Figs 6, 7 and 8, respectively. Average seawater temperatures at St.1 and St.2 were highest in Round 1 and lowest in Round 3 (Fig 6). At both sites, test plates immersed during the warmer seasons showed a tendency to have a larger fouled area. Specifically, test plates immersed during Round 4 (May 2014) showed the most fouling in terms of rankings. Barnacles, bryozoans and polychaetes occurred at St.1, while barnacles and bryozoans were the major fouling organisms at St.2. At both sites, the condition of fouling in the control group and on A-0 plates, which were coated with the paint containing 0 wt. % of Cu_2_O content, were almost the same. Trace amounts of biofouling were observed on A-3 plates with the paint containing 20 wt. % of Cu_2_O content, while no fouling was observed on plates coated with paints containing more than 30 wt.% of Cu_2_O content.

Hence, fouling on the plates at both experimental sites generally exhibited a similar tendency, which was a decrease in the degree of fouling with increasing Cu_2_O content in the paint. This tendency of decreasing degree of fouling with increasing Cu_2_O content in the paint was also observed in all rounds. The average rank of fouling for each experimental group of each site was calculated from values obtained from the four rounds.

## Discussion

### Concept of the laboratory experiment

In designing a laboratory experiment to assess the efficiency of AF paints, the authors took into consideration the following: (1), the condition of the test water; (2), the test organism; and (3), preparation of the test plates in a manner that simulated leaching from actual ships’ hull. In regard to the condition of the test water in bioassays, most literature available performed bioassays in a still water condition [27, 34, 52]. In most AF paints, antifouling agents leach from the paint surface and diffuse to the surrounding water. In a still water condition, the antifouling agent that leached out accumulates inside the experimental vessel. Thus, the concentration of the antifouling agent inside the vessel continuously increase with time. As a result, the concentration may increase to a level critical to the test organism, thereby making it difficult to appropriately assess the efficiency of the AF paint. In order to solve this problem, the authors designed a flow-through experimental system that allows the constant replacement of test water inside the test container. Mussels *M*. *galloprovincialis* were chosen as the test organism because it is a major macrofouler and a wide volume of literature is available on it [12].

Previous reports have assessed the efficiency of AF paints by quantifying byssus threads produced by mussels fixed on the surface of test plates and comparing the results between the control and experimental groups in a still water condition [31]. The production of byssus threads by mussels has been widely used as a standard for evaluating antifouling activity against mussels in inhibition assays. Moreover, young mussels were used as test organisms because they have higher byssus threads production activity as compared to adults [53]. The mussel byssus has numerous byssus threads, each formed within a groove in the mussel foot by a process resembling polymer injection-molding [54]. The byssus threads tether the mussel to the substratum via adhesive plaques that are linked to the stem root system embedded in the byssal gland of the foot [55, 56]. In this manner, mussels are able to regulate byssus threads production depending on the environmental condition via its foot imprinting behavior, which is temporarily affixing the tip of its foot to the substrate [57–59]. However, it is not yet clear if mussels can sense surfaces using their byssus system.

In general, newly sprayed AF paints are known to have higher leaching rates, and leaching stabilizes after a certain period of time, normally after 45 days under laboratory controlled condition [60, 61], but may vary depending on the type of paint [62, 63]. Official analytical methods are used to determine leaching rates of AF paints. However, rates derived from these methods may be overestimated, compared to actual leaching rates from ships’ hulls. As such, these overestimated rates can largely affect environmental risk assessment [64]. To ensure that leaching is stable during experiments, test plates were aged using a dynamic aging system [65]. This dynamic aging system simulated the stable leaching in actual vessels, and thus ensuring that biocide leaching from the AF paint has reached the steady state prior to the experiment.

#### The influence of exposure concentration of Cu_2_O on mussels

In this study, the repellant effect of AF paint on mussels was evaluated based on the production of byssus threads in a flow-through system. According to a toxicity study on mussels that was conducted in a still water condition, CuSO_4_ 5H_2_O was detected to be lethal from 250 mg/L, and mussel mortality was 68% at 500 mg/L [31]. The effect on byssus threads production was also detected from 250 mg/L [31]. The percentage of mussels that did not secrete byssus threads was 30% at 400 mg/L, and 90% at 500 mg/L [31]. In the group coated with the paint A-5, which contained 40 wt. % of Cu_2_O, the Cu_2_O concentration ranged from 22.4 to 42.2 μg/L after the experiment. This concentration range was 1/12,500 times lower than the value mentioned in the previous study [31]. Furthermore, no mussel mortality was observed after the 24-h experimental period. This result indicates that the rate of water exchange inside the tank effectively maintained the concentration of Cu_2_O in the test water to a level that was not lethal to the mussels.

#### Validation of the laboratory test as a standard method to evaluate the efficiency of AF paints

The quantification of byssus threads production of mussels in a flow-through system proved to be an expedient, applicable and indicative method for evaluating the efficiencies of paints containing Cu_2_O of varying concentrations, in terms of the equipment and methodology used, and the duration of the experiment. Differences in results observed between experiments indicate that byssus threads production of mussels vary among individuals and with the season. To minimize individual and seasonal variability of results, byssus threads production can be calculated as ratios in relation to their respective controls (Fig 5).

In the field, the leaching rate of Cu_2_O from a commercially available paint was more than 10 μg/cm^2^/day after 1year [66]. This leaching rate of Cu_2_O is well known as a standard limit value for antifouling [14]. This value is considered almost equivalent to the concentration of Cu_2_O in the test water of the group with 5 wt. % of Cu_2_O in this investigation in terms of the leaching rate. The present result obtained from the 5 wt. % Cu_2_O group is consistent with the standard limit value for antifouling, in that antifouling effect against the byssus threads production of mussel was observed at this concentration. Considering that only trace concentrations of Cu_2_O leached from the test panels during the experiments, the method described in this paper has higher sensitivity than in the previous investigation [31].

In the field experiment conducted in this study, biofouling on test paints gradually decreased with increasing content of Cu_2_O in the paints, and antifouling effect was observed at concentrations of 20 wt. % Cu_2_O and higher. On the other hand, results of laboratory bioassays showed that the inhibiting effect of Cu_2_O on byssus threads production was significant from 5 wt. % Cu_2_O (p < 0.001) and higher. Byssus threads production ratio for Cu_2_O at concentration of 10 wt. % and higher (p < 0.0001) decreased below 50%. From these results, byssus threads production of mussels clearly decreased at concentrations of 10 wt. % Cu_2_O and higher.

**Fig 9:**
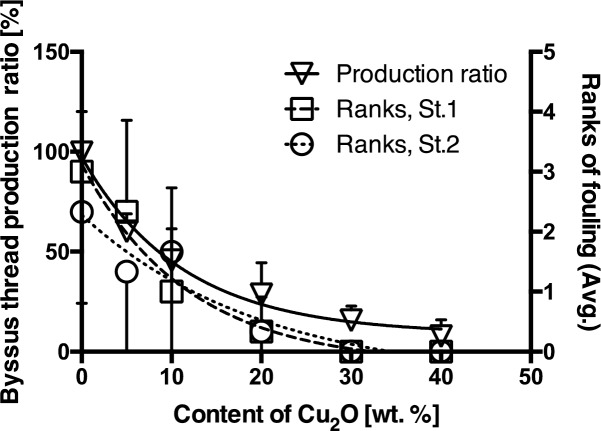
The relationship between byssus threads production ratio and average ranks of fouling in this study. The byssus threads production ratios calculated from laboratory bioassay (▽) and the ranks of fouling observed at St. 1 (□) and St. 2 (○) field tests plotted in relation to the concentration of Cu_2_O in the paints. The error bars on the open triangles indicates the SD in the ratio of byssus threads production.

To assess the antifouling property of the test paints, results of laboratory bioassay and field experiments were compared. The relationship between R and average ranks of fouling on test plates coated with varying concentrations of Cu_2_O are shown in Fig 9. Results of both laboratory and field experiments exhibited a similar tendency, where both the R and the average rank of fouling decreased with increasing concentration of Cu_2_O in the paint, while the relationship observed between the rank of fouling and Cu_2_O content in the paint was consistent at both stations (St.1: r^2^ = 0.9759, non-linear curve fitting, one-phase decay; St.2: r^2^ = 0.9135, non-linear curve fitting, one-phase decay). The purpose of this study was to develop this laboratory bioassay as a protocol for rapid initial testing of antifouling paint efficacy prior to field experiments. Biofouling data obtained from field experiments is affected by geographical and seasonal factors, and field experiments require a longer time (at least a one month test period) to evaluate the efficacy of antifouling paints [14]. On the other hand, the relationship observed in the laboratory bioassay between byssus threads production ratio and Cu_2_O content in the test antifouling paints clearly reflected the relationship observed in the field between the degree of fouling and the Cu_2_O content in the paint. Moreover, results in the former was obtained within 24 h. This indicates that the proposed method using mussels can be used as a sufficient verification test for the purpose of identifying efficient antifouling paints without having to conduct field experiments.

## Conclusion

A method for evaluating the efficacy of biocide-releasing AF paints was investigated in order to establish a reproducible and effective laboratory bioassay with a flow-through system using *M*. *galloprovincialis* as the test organism. Test paints with varying Cu_2_O content were prepared, and dynamic aging of test panels was also conducted to simulate the actual navigation state of marine vessels. Data with high reproducibility were obtained when byssus threads production of experimental groups were expressed as ratios of their control counterpart. A positive correlation was observed between the Cu_2_O content in the paints and the inhibition of byssus threads production. Field experiments showed antifouling efficacy at more than 20 wt.% of Cu_2_O content in the paint. Results of the field experiments were highly consistent with those of laboratory bioassays. In conclusion, a highly sensitive laboratory bioassay using mussels, which was established in this study, is an applicable method for universally evaluating the efficacy of AF paints. The proposed laboratory bioassay using the mussel, *Mytilus galloprovincialis*, is a valid method for evaluating general AF paints and can be used as a standard test method. Under proper flow conditions, this method can also be applied to test non-toxic foul-release coatings (FRC), such as silicone or PTFE-based FRC. The authors are currently developing a similar laboratory bioassay using barnacles and algae as test organisms.
